# Deep learning-based methods for natural hazard named entity recognition

**DOI:** 10.1038/s41598-022-08667-2

**Published:** 2022-03-17

**Authors:** Junlin Sun, Yanrong Liu, Jing Cui, Handong He

**Affiliations:** School of Resources and Environment, Anhui Agricultural University, Hefei, 230036 China

**Keywords:** Natural hazards, Computer science, Statistics

## Abstract

Natural hazard named entity recognition is a technique used to recognize natural hazard entities from a large number of texts. The method of natural hazard named entity recognition can facilitate acquisition of natural hazards information and provide reference for natural hazard mitigation. The method of named entity recognition has many challenges, such as fast change, multiple types and various forms of named entities. This can introduce difficulties in research of natural hazard named entity recognition. To address the above problem, this paper constructed a natural disaster annotated corpus for training and evaluation model, and selected and compared several deep learning methods based on word vector features. A deep learning method for natural hazard named entity recognition can automatically mine text features and reduce the dependence on manual rules. This paper compares and analyzes the deep learning models from three aspects: pretraining, feature extraction and decoding. A natural hazard named entity recognition method based on deep learning is proposed, namely XLNet-BiLSTM-CRF model. Finally, the research hotspots of natural hazards papers in the past 10 years were obtained through this model. After training, the precision of the XLNet-BilSTM-CRF model is 92.80%, the recall rate is 91.74%, and the F1-score is 92.27%. The results show that this method, which is superior to other methods, can effectively recognize natural hazard named entities.

## Introduction

Natural hazards will cause huge casualties, property losses and economic damage to human beings^1–3^. Recognition of natural hazard text information can facilitate the reuse of natural hazard literature and provide the reference for natural hazard mitigation. With the development of computer technology, research on natural language understanding and text data mining is deepening, and it is increasingly important to recognize the disaster information in natural hazard text efficiently and accurately^4–7^. Named entity recognition (NER)^8^ is a technique for extracting the words or expressions of a specific entity from unstructured text data. It was first defined by the message understanding conference (MUC)^9^ into three categories (entity type, time type and number type) and seven subcategories (person name, institution name, place name, time, date, currency and percentage)^10^. Named entity recognition is an important foundation of natural language processing tasks^11^, such as information extraction, question answering systems, knowledge mapping and machine translation. The purpose of named entity recognition is to recognize and classify the components representing named entities in a text. Natural hazard named entity recognition (NHNER) is a technique for identifying natural hazard entities from large numbers of natural hazard texts. The purpose of NHNER is to identify important disaster information from natural hazard texts and classify the identified content into predefined semantic categories to support data analysis and natural hazard management.

Early named entity recognition used rules and dictionary methods^12^. Methods based on rules and dictionaries use artificial construction rule extraction to find a matching string from the text, including the DL-Co Train^13^, automatic rule generation^14^, and LaSIE-II^15^ methods. When the extraction rules accurately reflect the linguistic phenomena, these methods can achieve high recognition for a specific corpus. The disadvantage is that the improvement in the recognition effect relies on many rules, is extremely dependent on artificial features, and that the rule method is complex^16^. Machine learning methods have been applied to natural hazard information extraction^17^. Methods based on machine learning are trained by manually tagging the corpus, which is realized by recognizing the boundary of the named entity and then classifying or serially tagging each word. This class of methods mainly includes the hidden Markov model (HMM)^18^, maximum entropy (ME)^19^, maximum entropy Markov model (MEMM)^20^, support vector machine (SVM)^21^, and conditional random fields (CRF)^22^. The advantage of these methods is that they can be transplanted to a new field with few or no changes, and the new corpus can be trained once. The disadvantages are that it is difficult to extract features, there is heavy reliance on the corpus, and there are few general corpora.

In recent years, the deep learning method based on word vector features has been widely used in natural hazard named entity recognition and has achieved good results on most corpora^23,24^. Methods based on the word vector feature of deep learning are derived from the deep learning technology associated with a neural network. It uses a word vector to represent words and then divides the word vector into different entity classes. Finally, it automatically obtains word features through text expression. Compared with the earlier dictionaries, rules and machine learning methods, the deep learning method can solve the problem of data scarcity in high latitude vector space, and the word vector contains more semantic information than the artificial feature^25^. In terms of natural hazard named entity recognition, researchers have explored numerous methods based on deep learning, such as a deep learning classifier algorithm based on RNN was used to evaluate the Nepal earthquake data set^26^; a neural network algorithm having attention-based bidirectional long short-term memory with a conditional random field layer (Att-BiLSTM-CRF) was used to monitor Natural Disaster Social Dynamics^27^; and a multi-branch bidirectional gated recurrent unit (BiGRU) layer and a conditional random field (CRF) model was used to recognize named entities of geological hazards^28^. This kind of method automatically learns features and trains a sequence annotation model with the help of a neural network. Its performance exceeds those of the traditional methods based on artificial features. This is one of the current research hotspots. In this paper, natural hazard named entity recognition methods based on deep learning are compared based on the following three aspects: (1) pretraining methods; (2) feature extraction methods; (3) decoding methods.

The pretraining method uses a large-scale unlabeled text corpus to train the deep network structure, which is called the "pretraining model", to obtain the word vector. The pretraining model was the static method Word2Vec in the early stage, and then many dynamic methods were proposed, such as BERT^29^, ALBERT^30^ and XLNET^31^. These models can dynamically adjust the expression of the text according to the context. After adjustment, they can better express the specific meaning of the word in the context and effectively solve the problem of polysemy. XLNet has the following advantages: 1. It can learn the language structure from the rules of corpus; 2. More refined semantic modeling: XLNet is currently the most refined model for semantic modeling from "one-way" semantics to "two-way" semantics, from "short-range" dependencies to "long-range" dependencies; 3. When the model capacity is large enough, the logarithm of data volume is close to proportional to the performance improvement within a certain range. Compared with BERT and ALBERT models, XLNet uses autoregressive language model to solve the problem of independent prediction between words, and permutation language model is used to obtain true bidirectional context information from autoregressive model^32^.

Feature extraction mainly transforms the input word vector, learns the vector representation of contextual information, and extracts the semantic information of sentences. Feature extraction is generally implemented by a coding layer in the NER framework. BiLSTM^33^ and BiGRU^34^ models are commonly used for feature extraction. BiLSTM uses two-layer LSTM to obtain the forward and backward information of text sequences and splicing them to obtain the final hidden layer feature representation, which can solve the problem of capturing contextual semantic information and effectively improve the effect of named entity recognition^35^.

The decoding method is the last step in the NER framework, namely, the decoding layer. The decoding method is used to predict the natural hazard label corresponding to each word in the text. At present, the most commonly used decoding methods include CRF. Through the CRF, the label sequence with the highest global probability can be output to complete the recognition of entities.

In this paper, a named entity annotated corpus for natural hazard is constructed with respect to the following aspects: collection principle and collection of the corpus, construction of annotation system and named entity classification, and consistency evaluation of the annotated corpus. This paper studies the methods of natural hazard named entity recognition based on deep learning, compares the advantages and disadvantages of each pretraining method in NHNER through experiments, and combines each pretraining method, feature extraction method and decoding method, so as to obtain the optimal natural hazard named entity recognition method. Finally, a natural hazard named entity recognition method based on XLNet-BiLSTM-CRF model is proposed. XLNet-BilSTM-CRF model uses pretrained language model vector to replace the traditional static word vector with dynamic words trained in large-scale corpus to serialize the natural hazard text, so as to effectively solve the problem of polysemy, and make the semantic representation of context more accurate. The generalized autoregressive prediction model XLNet can make up for the non-independent prediction of BERT model. BiLSTM can capture the long-distance dependent features in natural hazard text, and finally CRF can ensure the correctness of label sequence^36^. XLNet- BilSTM-CRF uses a neural network to automatically mine the hidden features of text, reduces the dependence on manual rules, and realizes the task of natural hazard named entity recognition efficiently and accurately. And the popular research topics of natural hazard papers in recent 10 years are detected through this model.

## Methods

### Construction of NHNER corpus

This paper constructs the corpus from the following three aspects: 1. Collection principle and collection of the corpus; 2. Construction of annotation system and named entity classification; and 3. Consistency evaluation of the annotated corpus.

### The collection principle and collection of the corpus

Corpus collection follows the following two principles: 1. Scientific sampling and random sampling ensure that the corpus is objective, comprehensive and balanced; and 2. The corpus contains rich natural hazards information to ensure that the number natural hazard named entities is sufficient for training and testing.

In this paper, the papers related to natural hazards in Wanfang Database is taken as the collection object. To ensure the scientific nature of the experiment, the abstract of natural hazards paper are collected as samples, and the samples are preprocessed. Preprocessing includes removing a series of nontext data such as pictures, spaces and tables and deleting special symbols and sentences irrelevant to natural hazards. Table 1 lists detailed information about corpus collection.

**Table 1:** Details of corpus sources.

Corpus name	Details of hazard category	words	sentences
Natural hazard annotation corpus	Earthquake	23,411	241
	Tsunami	7,695	69
	Coastal erosion	5,887	41
	Landslide	19,517	105
	Meteorological extreme events	34,926	188
	Flood	9,956	56
	Soil erosion and desertification	6,408	47
	Wildfires	8,542	59

### Construction of annotation system and named entity classification

In this paper, the annotation system is determined before labeling and remains unchanged in the labeling process to ensure the consistency of labeling. By comprehensively referring to the relevant classic literature in natural hazards^37–40^ and the annotation characteristics of NHNER, this paper formulates the following annotation system:

1 $$Haz\_sentence \, Model=(origin,Loc,Haz,Met)$$

In the above formula, Haz_sentenceModel is a natural hazard sentence annotation model developed in this paper. Origin represents the original statement. Loc, Haz and Met represent the geographical location, natural hazards and research method, respectively. The explanation and value range of each variable in the natural hazard sentence annotation model are shown in Table 2. According to the annotation system, natural hazard named entities are divided into three categories: geographical location, natural hazards and research method.

**Table 2:** Description of variables in the annotation system.

Variable	Explanation	Value range
Loc	Geographical location	1. Physical geographical location, such as the Pacific or Himalayas 2. Cultural geographical location, such as province, city, or county
Haz	Natural hazards	Coverage includes such categories of hazard as meteorological extreme events, storm surges, tsunamis, floods, landslides, erosion, earthquakes, volcanoes, soil erosion and desertification
Met	Research method	Methods, techniques and models

### Consistency evaluation of annotated corpus

Four authors of this paper with experience in Earth science and NER were used as annotators to annotate the text. These four annotators were divided into two groups with two people in each group. Each annotator needed to annotate the original corpus once.

Unified annotation standards can effectively reduce the differences between various annotators and reduce errors and inconsistencies in corpus annotation. The inter-annotator agreement is as follows:

1. There are three types of named entities: geographical location, natural hazards and research method.
2. Annotations follow the principle of nonoverlapping, nonnested, and nonstopping punctuation marks (such as commas, periods, and pause) in named entities.
3. In the case of inconsistent labels, it is necessary to refer to the percentage of overlap selection among all annotators and to select labels with a high overlap rate.
4. The annotated words must be related to natural hazards and cannot deviate from the basis of natural hazards.

Annotation consistency can usually be expressed by two indicators: the kappa value^41^ and the F1-score^42^. The kappa value is generally used for annotation evaluation of positive and negative cases, such as corpus annotation of emotion classification. In the annotation of the entity recognition corpus, the unmarked words can be regarded only as negative examples and are difficult to count. When there are many negative cases that are difficult to count, the F1-score can be used for evaluation. In this paper, the consistency of corpus annotation is evaluated by the F1-score. The specific method regards the annotation results of an annotator A1 as the standard answer and calculates the precision (P), recall (R) and F1-score of the annotation results of another annotator A2. The calculation formula is shown in formulas 2–4.

2 $$P=\frac{consistent \,  annotation  \, results  \, of \, {A}_{1}  \, and \,  {A}_{2} }{{ label \,  results  \, of  \, A}_{2}}$$

3 $$R=\frac{consistent  \, annotation  \, results  \, of  \, {A}_{1}  \, and \,  {A}_{2} }{{ label  \, results  \, of  \, A}_{1}}$$

4 $$F1=\frac{2*R*P}{R+P}$$

### Pretraining method

The pretraining method uses large-scale unlabeled natural hazard corpora to train the deep network structure, which is called the "pretraining model", to obtain the word vector. The pretraining language model provides a dynamic pretraining technique, that is, a context-dependent text representation, which can effectively process polysemy. In this paper, the BERT, ALBERT and XLNet pretraining models are used to study the application effect in the natural hazard named entity recognition.

### BERT

Bidirectional encoder representation from transformers (BERT) is a pretrained language model^43^. Among these transformers, the algorithm framework can capture the bidirectional relationship in words and sentences^44^. When entering the natural hazard text, each word in the sentence is calculated with other words by an attention calculation formula. The calculation formula of attention is shown in formula (5). By calculation, information about sentences can be captured from the connections between words.

5 $$Attention\left(Q,K,V\right)=softmax\left(\frac{Q{K}^{T}}{\sqrt{{d}_{k}}}\right)V$$

In formula (5), Q, K, and V represent the word vector matrix of natural hazard text, and dk is the embedding dimension.

**Figure 1:**
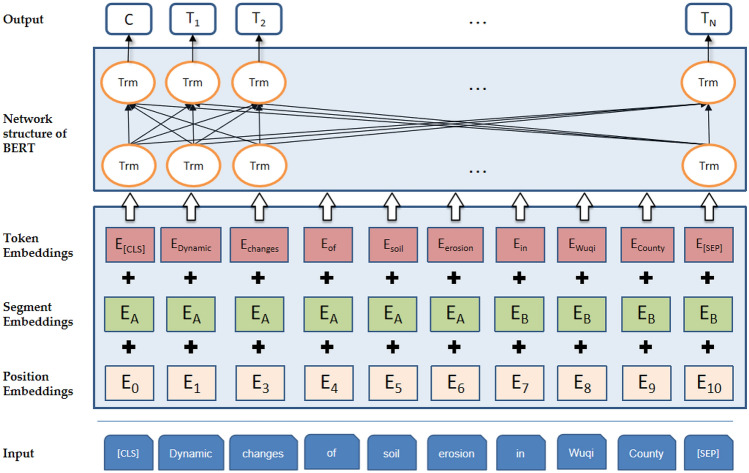
The overall structure of the BERT model.

The overall structure of the BERT model is shown in Fig. 1. In Fig. 1, En is the encoded representation of words, Trm is the transformer structure, and Tn is the trained word vector. The BERT model takes natural hazard text as input. The token embedding separates input words into different tokens and adds two special symbols, [CLS] and [SEP], to indicate the beginning of the text instance and the end of the sentence. Segment embedding is used to distinguish two sentences. Position embedding represents the location information of a word. The input word vector of BERT is obtained by adding three vectors: token embedding, segment embedding and position embedding.

### ALBERT

ALBERT is a lite BERT, which has fewer parameters and results in effects similar to those of BERT. BERT is often limited by hardware memory in practical applications because of its large number of parameters^45^. In this paper, an ALBERT model with 60 M parameters is used, which is much smaller than BERT's 110 M parameters. ALBERT mainly made improvements in the following two aspects: first, ALBERT adopted the method of factorized embedding parameterization and cross-layer parameter sharing to reduce the number of parameters, on the one hand reducing the number of parameters, and on the other hand effectively improving the stability of the model. Secondly, ALBERT proposed a sentence-order prediction (SOP). The SOP keeps the positive sentence relationship unchanged and has the correct context order during training; for negative sentence relations, SOP reverses the sentence order to input a pair of natural hazard sentences into the model and allows the model to predict the sequence of the two sentences. This approach focuses on the coherence between natural hazard sentences and prevents the theme from being affected.

### XLNet

XLNET is a generalized autoregressive method, which realizes bidirectional context information prediction based on a traditional autoregressive language model^46^. XLNet uses the Permutation Language Model (PLM), whose core idea is to rearrange the input sequence through the Attention Mask matrix in Transformer and realize the bidirectional prediction by learning the sequence feature information of different sorts. Meanwhile, the original word order is not changed, and the problem of information loss under the Mask mechanism in the BERT model is effectively optimized.

**Figure 2:**
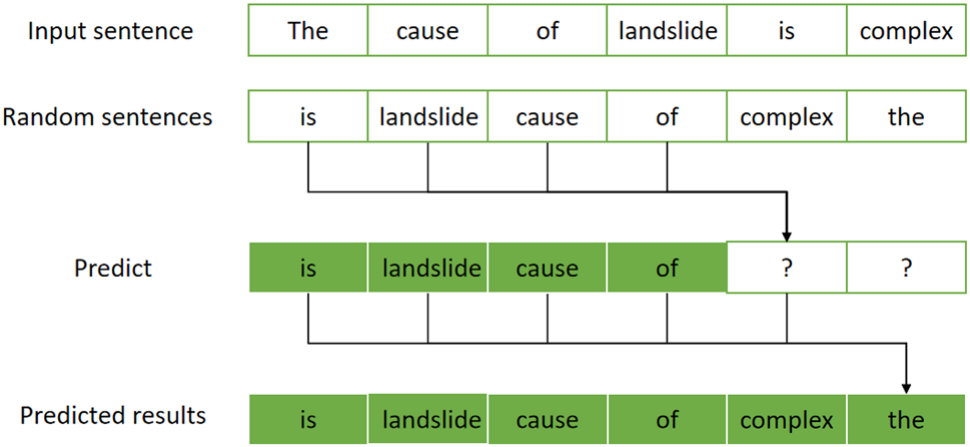
PLM mechanism demo diagram.

The PLM mechanism of XLNet is shown in Fig. 2. When the model input sentence is "The cause of landslide is complex", and a group of sequences randomly generated in Transformer are "is landslide cause of complex the ", then the rearranged word "complex" can reflect the information of the preceding words and enable predictions based on the preceding words. The last word " the" can enable predictions based on all the information in the sentence. This allows the predicted words to predict contextual words within Transformer. XLNet applies the recurrence mechanism and relative position encoding based on Transformer structures. XLNet inserts hidden information between segments through the recurrence mechanism, and the later segments can use the information of the earlier segments to realize the transmission of natural hazards information.

### Feature extraction method

Feature extraction mainly transforms the input word vector, learns the vector representation of contextual information, and extracts the semantic information of sentences. In this paper, the BiLSTM and BiGRU models are selected to study application effects in the NHNER.

### BiLSTM

Long short-term memory (LSTM) is a kind of time-cycling neural network that can protect and control the state of neural units by effectively utilizing the dependence of long-distance sentences through a gating mechanism^47^. The LSTM unit protects and controls the memory (or forgetting) state of the neural network unit with respect natural hazard information through three structures (natural hazard information forgetting gate, natural hazard information input gate and natural hazard information output gate). The formulas of the LSTM gate mechanism are shown in formulas 6–10.

6 $${I}_{t}=\sigma ({a}_{xI}{x}_{t}+{a}_{hI}{h}_{t-1}+{a}_{cI}{C}_{t-1}+{b}_{I})$$

7 $${F}_{t}=\sigma ({a}_{xF}{x}_{t}+{a}_{hF}{h}_{t-1}+{a}_{cF}{C}_{t-1}+{b}_{F})$$

8 $${C}_{t}={F}_{t}{C}_{t-1}+{i}_{t}\mathit{tan}h({a}_{x}C{x}_{t}+{a}_{hC}{h}_{t-1}+{b}_{C})$$

9 $${O}_{t}=\sigma ({a}_{xO}{x}_{t}+{a}_{hO}{h}_{t-1}+{a}_{CO}{C}_{t}+{b}_{O})$$

10 $${H}_{t}={O}_{t}\mathit{tan}h({C}_{t})$$

In the above formulas, ${I}_{t}$ is used to judge whether to update the unit status with the current input, which represents the natural hazard information input gate. ${F}_{t}$ outputs a value between 0 and 1 to judge whether to forget the memory of the previous time point, which represents the natural hazard information forgetting gate. ${O}_{t}$ is used to output memory information, which represents the natural hazard information output gate. The values a and b represent the weight and bias of the calculation natural hazard information input gate, natural hazard information forgetting gate and natural hazard information output gate, which can be updated in the training. ${C}_{t}$ represents the updated state of the neural unit at time t, and ${x}_{t}$ represents the input variable at time t. $\sigma$ stands for the sigmoid function, and $tanh$ stands for the hyperbolic tangent function.

**Figure 3:**
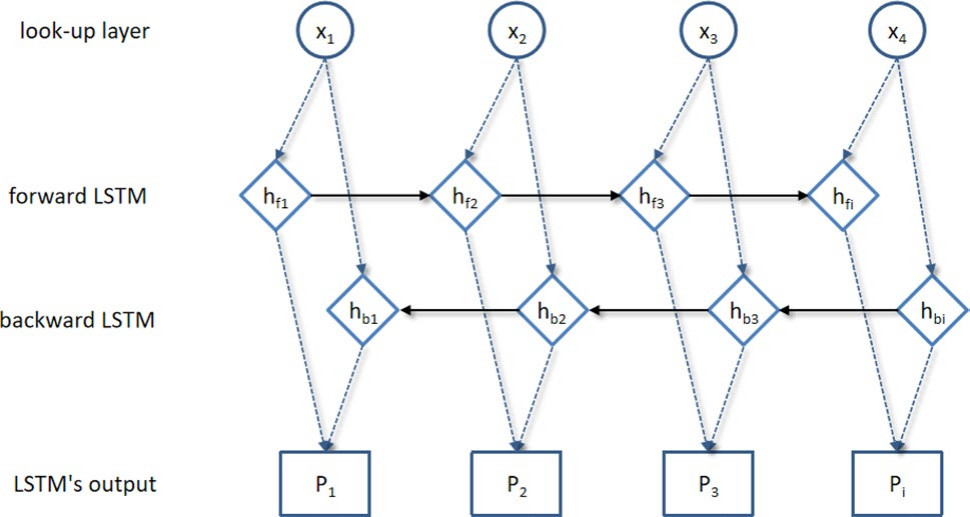
BiLSTM model structure diagram.

BiLSTM is a bidirectional LSTM model, that is, a neural network that combines forward LSTM and backward LSTM. Through two-way propagation, BiLSTM can obtain the coding information from back to front and capture the context relationship through two-way coding^48^. The structure of the BiLSTM model is shown in Fig. 3.

Model BiLSTM receives the word vectors trained by model BERT through the look-up layer, and the input word vectors are operated forward and backward in forward LSTM and backward LSTM, respectively^49^. Then, the forward hidden vector (hfi) and backward hidden vector (hbi) of a word vector (Xi) are spliced through LSTM’s output layer to obtain a complete feature vector (Hi), as shown in formula (11). Finally, the predicted score (Pi) of the label corresponding to each input data point can be calculated by formula (12).

11 $${H}_{i}=[\overrightarrow{{h}_{fi}}\cdot \overleftarrow{{h}_{bi}}]$$

12 $${P}_{i}=tanh(W\cdot {H}_{i})$$

In formula (12), the weight matrix W is the parameter of the model to be learned in training.

### BiGRU

BiGRU is a special kind of recurrent neural network. Like BiLSTM, BiGRU is designed to solve the problem of RNN long-term memory and vanishing back-propagated gradients. BiGRU combines BiLSTM's natural hazard information forgetting gate and natural hazard information input gate into a natural hazard information update gate, which is a simpler network model. The formulas of the BiGRU gate mechanism are shown as formulas 13–16.

13 $${r}_{t}=\sigma ({w}_{rx}{x}_{t}+{w}_{rh}{h}_{t-1}+{b}_{r})$$

14 $${z}_{t}=\sigma ({w}_{zx}{x}_{t}+{w}_{zh}{h}_{t-1}+{b}_{z})$$

15 $$\stackrel{\sim }{{h}_{t}}=\mathit{tan}h({w}_{xh}{x}_{t}+{r}_{t}\otimes {w}_{hh}{h}_{t-1})$$

16 $${h}_{t}=\left(1-{z}_{t}\right)\otimes {h}_{t-1}+\stackrel{\sim }{{h}_{t}}\otimes {z}_{t}$$

In the above formulas, ${r}_{t}$ represents the natural hazard information reset gate, ${z}_{t}$ represents the natural hazard information update gate, w is the weight matrix, b is the bias, and ⨂ represents the Hadamard product.

### Conditional random field

A CRF is a sequence labeling algorithm^50^. Considering the correlation between tags, we use a CRF to determine the tag sequence. A natural hazard text $X\left({x}_{1}{,x}_{2},\dots ,{x}_{n}\right)$ produces a predicted sequence of natural hazard labels $L\left({l}_{1}{,l}_{2},\dots ,{l}_{n}\right)$, and formula (17). ^51^ can indicate the score of the sequence L.

17 $$S\left(X,L\right)=\sum_{i=0}^{n}{A}_{{l}_{i},{l}_{i+1}}+\sum_{i=1}^{n}{P}_{i,{l}_{i}}$$

In formula (17), S represents the evaluation score of the natural hazard label sequence, matrix A is the transfer matrix, Ai,j represents the probability of transferring from natural hazard label i to natural hazard label j, l is the mark of the natural hazard label sequence, and n is the sequence length. Pi,j is the probability of the jth natural hazard tag of the ith word in the sentence^52^. $S\left(X,L\right)$ is equal to the sum of the scores of all the words in the sentence, and each score is composed of the transfer score matrix A and the score matrix P.

The softmax function is used to normalize probability, as shown in formula (18). $\tilde{L }$ represents the authenticity of the natural hazard label sequence, and ${L}_{X}$ represents all possible natural hazard label sequences.

18 $$p\left(L\left|X\right.\right)=\frac{{e}^{S\left(X,L\right)}}{{\sum }_{\tilde{L }\in {L}_{X}}{e}^{S\left(X,\tilde{L }\right)}}$$

In the decoding process, the output sequence with the maximum score is predicted by formula (19), which is used as the final natural hazard labeling result.

19 $${L}^{*}={argmax}_{\tilde{L }\in {L}_{X}}S\left(X,\tilde{L }\right)$$

In the CRF layer, s is used to evaluate the probability of the natural hazard label sequence, the label sequence with higher accuracy can be obtained through the evaluation score, and the predicted tag is legal to reduce the probability of prediction error.

### XLNet-BiLSTM-CRF

In this paper, a natural hazard named entity recognition model based on XLNet-BiLSTM-CRF is proposed. XLNet-BiLSTM-CRF model is divided into three parts. In the first part, the text of natural hazards is input into XLNet layer, and words are encoded by XLNet and transformed into word vectors to obtain vectors with natural hazards characteristics, which are extracted from natural language^53^. In the second part, after obtaining the word vector representation of each sentence, the word vector sequence is input to the BiLSTM as the input data. Then, the BiLSTM is used to encode the vectors in two directions to increase the relevance between contexts and to provide complete natural hazards information on sequence points for the output layer^54^. BiLSTM pays attention to local relationship and location information. Location information of XLNet is realized through location coding, which may become weak after multi-layer forward transmission. In this case, training effect will be better through BiLSTM supplement. In the third part, CRF is used to decode and output the natural hazard label sequence with the highest global probability. The XLNET-BilSTM-CRF model uses pretrained language model vectors. The natural hazard text is serialized by using dynamic words trained in corpus to replace the traditional static word vector. This can effectively solve the problem of polysemy and make the semantic representation of context more accurate. This model uses a neural network to automatically mine the hidden features of text, reduces the dependence on manual rules, and realizes the task of natural hazard named entity recognition efficiently and accurately.

## Experiment

### Experimental data and experimental setup

The papers related to natural hazards in Wanfang Database is selected to con-struct a corpus. In addition, an F1-score of 86.68% is obtained through annotation con-sistency evaluation. In this paper, the dataset is divided by random extraction into three sets: 80% consists of the training set, 10% forms the validation set and 10% is used as the test set. We have made the natural hazard corpus public. Table 3 shows the details of the corpus.

**Table 3:** Corpus details.

Corpus and website	Label			Word number
	Loc	Haz	Met	
Natural hazard corpus	930	1,637	754	116,342
Open source website	https://github.com/SunJunl/Natural-hazards-NER-corpus			

**Figure 4:**
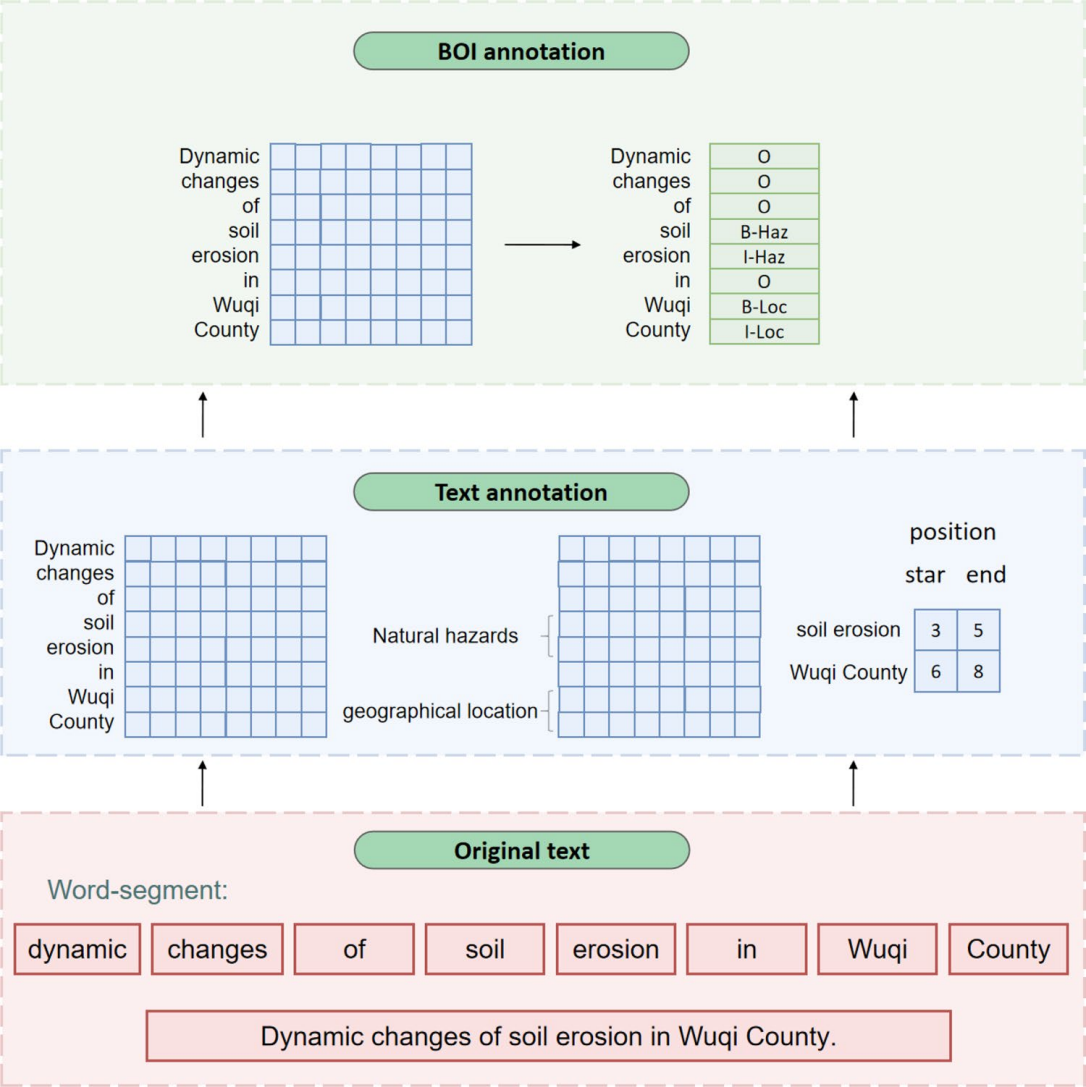
Labeling process.

This experiment is annotated according to the annotation system constructed in part 2.1 above. We use Python language to apply BOI labels to the labeled data. In this labeling method, B means beginning, representing the initial character of a natural hazard entity; I means inside, representing the noninitial component of the natural hazard entity; and O means outside, indicating that the marked text does not belong to the natural hazard entity. The label of geographical location is defined as Loc, the label of natural hazards is defined as Haz, and the label of research method is defined as Met. The annotation process is shown in Fig. 4. The original text is manually annotated to obtain the entity, entity category, starting position and ending position, and then transformed verbatim into the corresponding BOI annotation.

This paper uses web crawler technology to retrieve papers published from 2010 to 2020 and related to natural hazards. Crawling technology is mainly realized by BeautifulSoup and requests. We return the crawled content to a text file, which contains the title and abstract of each paper. This paper crawls 12,387 papers, totaling 208,890 characters, from the Wanfang Database. These data are recognized by the model proposed in this paper to analyze the research status of natural hazards in the last ten years.

This paper uses the TensorFlow (version 1.13.1) deep learning framework and Python (version 3.7.1) programming language to establish the experimental model. Many parameters are involved in the model training of deep learning. We attempt to fine-tune the parameters many times and obtain the most suitable parameters with the highest performance. In this paper, the initial learning rate is set to 0.0005, the maximum sequence length is set to 128, the warmup proportion is set to 0.1, the batch size is set to 64, and the number of epochs is set to 40. The dropout rate is set to 0.5, which is a way to prevent the neural network from overfitting.

### Deep learning method selection and evaluation criteria

In this paper, the method of adding CRF into the pretraining model was selected to compare the performance of different pretraining models, namely BERT-CRF, ALBERT-CRF and XLNet-CRF.

Some of the most advanced named entity recognition methods (which have not been used by NHNER) are applied to the corpus constructed in this paper to analyze the performance of these models.

This paper selects 9 models as the research objects of this experiment, including: (1) BERT-CRF, (2) ALBER-CRF, (3) XLNet-CRF, (4) BERT-BiLSTM, (5) BERT-BiLSTM-CRF, (6) ALBER-BiLSTM-CRF, (7) XLNet-BiLSTM-CRF, (8) BERT-BiGRU-CRF, (9) ALBERT-BiGRU-CRF, (10) XLNet-BiGRU-CRF, (11) BiGRU-CRF, and (12) BiLSTM-CRF.

**Figure 5:**
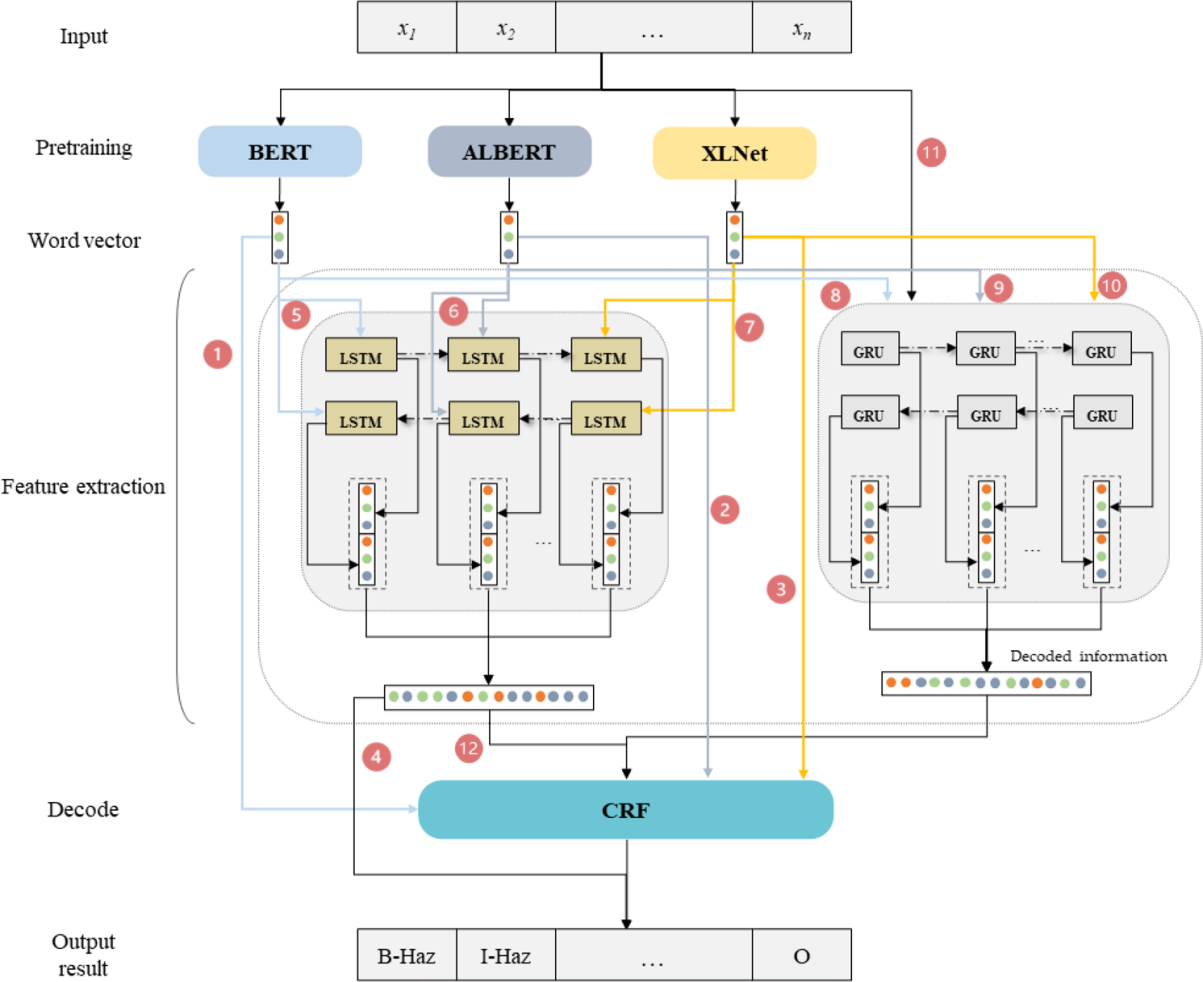
Model selection and framework: (1) BERT-CRF, (2) ALBER-CRF, (3) XLNet-CRF, (4) BERT-BiLSTM, (5) BERT-BiLSTM-CRF, (6) ALBER-BiLSTM-CRF, (7) XLNet-BiLSTM-CRF, (8) BERT-BiGRU-CRF, (9) ALBERT-BiGRU-CRF, (10) XLNet-BiGRU-CRF, (11) BiGRU-CRF, (12) BiLSTM-CRF.

In this paper, methods (1), (2) and (3) are used to study the performance, advantages and disadvantages of the pretraining model. Methods (8) and (9) are compared to study the performance, advantages and disadvantages of feature extraction methods. Comparison of two groups of models, (1–3) and (5–7), enables study of the influence of adding feature extraction methods on model performance. Comparison of (4) and (5) enables study of the influence of the decoding layer on model performance. The selection and framework of the model is shown in Fig. 5.

In this paper, the precision (P), recall (R) and average value (F1-score) are used as evaluation indices to test the performance of different models. The calculation formulas for the three evaluation indices are shown as formulas 20–22.

20 $$P=\frac{TP}{TP+FP}$$

21 $$R=\frac{TP}{TP+FN}$$

22 $${F}_{1}=\frac{2*R*P}{R+P}$$

In the above formulas, TP is the number of correctly predicted natural hazard entities, FP is the number of predicted natural hazard entities but non entities, and FN is the number of predicted non entities but are natural hazard entities.

### Comparison of the performance of different pretraining models

In the training of the pretraining model, the requirements of the corpus and server are relatively high, and the training time is very long. Therefore, we use the pretraining models already published by BERT, ALBERT and XLNet, including "BERT_base_Chinese", "ALBERT_base_zh" and "Chinese_XLNet_base". These pretrained models can be fine-tuned with a single additional output layer on a task-specific dataset. We only need to fine-tune the parameters (such as the training batch size) to use these models for NHNER tasks. We tune the hyperparameters of this fine-tuning step using the files from our validation set. The resulting parameters are as follows: learning rate of 0.0005, batch size of 64, and epoch number of 40. After pretraining the model, we link CRF decoding to predict the natural hazard labelling sequence, and this can improve the performance of the model. Table 4 shows the results of the evaluation.

**Table 4:** Evaluation results of pretraining model.

Model	P	R	F1
BERT-CRF	85.99	86.10	86.00
ALBERT-CRF	87.70	89.82	88.75
XLNet-CRF	91.00	91.73	91.36

## Results and discussion

### Performance analysis of deep learning models

In this paper, the training results of all models in the above experiments are sorted and analyzed. These include: (1) BERT-CRF, (2) ALBER-CRF, (3) XLNet-CRF, (4) BERT-BiLSTM, (5) BERT-BiLSTM-CRF, (6) ALBER-BiLSTM-CRF, (7) XLNet-BiLSTM-CRF, (8) BERT-BiGRU-CRF, (9) ALBERT-BiGRU-CRF, (10) XLNet-BiGRU-CRF, (11) BiGRU-CRF, and (12) BiLSTM-CRF. The training results include the precision (P), recall (R) and F1-score (F1) of each model. The specific results are shown in Table 5.

**Table 5:** Performance of nine deep learning models on natural hazard corpus.

Deep learning model	Evaluation	Natural hazard corpus			Weighted avg	Time/s	Mean F1-socre for five runs
		Loc	Haz	Met			
BERT-CRF	P	87.25	85.24	85.48	85.86	690	86.16
	R	82.46	89.65	86.19	86.85		
	F1	84.79	87.39	85.83	86.35		
ALBER-CRF	P	88.41	87.57	87.12	87.70	402	88.59
	R	90.05	90.16	89.24	89.92		
	F1	89.22	88.85	88.17	88.79		
XLNet-CRF	P	90.90	91.77	90.34	91.20	549	91.13
	R	90.27	92.00	92.93	91.73		
	F1	90.58	91.88	91.62	91.46		
BERT-BiLSTM	P	78.47	81.54	75.01	79.20	721	79.58
	R	76.25	82.38	79.89	80.10		
	F1	77.34	81.96	77.37	79.65		
BERT-BiLSTM-CRF	P	83.92	89.36	81.25	86.00	763	86.46
	R	88.57	82.41	92.86	86.51		
	F1	86.18	85.74	86.67	86.45		
ALBER-BiLSTM-CRF	P	89.61	86.23	85.85	87.09	423	88.93
	R	88.77	90.58	90.37	90.03		
	F1	89.19	88.35	88.05	88.94		
XLNet-BiLSTM-CRF	P	93.89	92.43	92.28	92.80	681	92.25
	R	92.33	91.58	91.37	91.74		
	F1	93.10	92.00	91.82	92.27		
BERT-BiGRU-CRF	P	83.06	86.81	83.62	85.04	1458	86.74
	R	87.34	86.66	92.84	88.25		
	F1	85.15	86.73	87.98	86.62		
ALBER-BiGRU-CRF	P	86.24	89.58	86.01	87.83	953	88.27
	R	89.31	89.44	89.35	89.38		
	F1	87.74	89.51	87.65	88.60		
XLNet-BiGRU-CRF	P	91.12	91.08	92.69	91.46	1007	91.62
	R	91.99	92.00	91.25	91.83		
	F1	91.55	91.53	91.96	91.64		
BiGRU-CRF	P	75.86	87.16	81.43	82.69	517	80.84
	R	77.14	80.36	83.33	80.13		
	F1	76.49	83.62	82.37	81.39		
BiLSTM-CRF	P	71.15	79.28	86.71	78.69	416	80.53
	R	74.30	86.43	85.22	82.76		
	F1	72.69	82.70	85.96	80.67		

From the perspective of the pretraining model, the three pretraining models are all 12-layer structures, and hidden is 768. The difference among them lies in the size of parameters, BERT, ALBER and XLNet parameters are 110 M, 12 M and 117 M respectively. We can determine from the experimental results of BERT-CRF, ALBER-CRF and XLNet-CRF that AlBERT has the fastest training speed, and BERT and ALBERT have similar performance, with 2.43% F1-score difference. Due to the difference in the size of parameters, the pretraining model with small number of parameters can obtain lower computational cost and faster training time. The F1-score of the XLNet model is 91.13%, which achieves the best effect and the training time is suitable for the task of this paper, indicating that XLNet can improve the performance of the model by adopting an autoregressive language model to solve the prediction independence between words.

From the perspective of the feature extraction method, we set the same number of parameters for BiLSTM and BiGRU to ensure fair comparison. The parameter information: the number of hidden layer nodes of BiLSTM and BiGRU is 128, the maximum sequence is 128, dropout is 0.5, the initial learning rate is 0.0005, the warmup proportion is 0.1, the batch size is 64, and the number of epochs is 40, and Adam is used as the optimizer. We can see from the experimental results of BiGRU-CRF and BiLSTM-CRF that the F1 of the BiGRU model is 0.31% higher than that of the BiLSTM model, but the training time is 101 s slower than that of BiLSTM model. Compared with the model without feature extraction, BERT-BiLSTM-CRF, ALBER-BiLSTM-CRF and XLNet-BiLSTM-CRF increased by 0.3%, 0.34% and 1.16%, respectively. For the structure of the existing pretraining model, feature extraction can improve the performance of the model. It can be seen from the experimental results of BERT-BiLSTM-CRF, ALBER-BiLSTM-CRF, XLNet-BiLSTM-CRF, BERT-BiGRU-CRF, ALBERT-BiGRU-CRF and XLNet-BiGRU-CRF, the BiLSTM and BiGRU have similar performance (BERT-BiGRU-CRF is 0.28% higher than BERT-BilSTM-CRF, ALBERT-BilSTM-CRF is 0.66% higher than ALBERT BiGRU-CRF, XLNet-BilSTM-CRF is 0.63% higher than XlNet-BiGRU-CRF), but the training time of BiGRU is significantly higher than that of BiLSTM (training time difference is: 695 s, 530 s, 326 s), which indicates that it consumes high computing cost.

From the perspective of the decoding layer, we can see from the experimental results of BERT-BiLSTM and BERT-BiLSTM-CRF that the difference in F1 score between them is 6.88%. BERT-BiLSTM using the CRF method is superior to BERT-BiLSTM without the CRF method. Adding the CRF layer helps to improve model performance because it captures dependencies between natural hazard labels.

From the overall point of view, the XLNet-BiLSTM-CRF model achieves the best performance, with the highest overall score, and the F1 score is 92.25%, and the training time was suitable for the natural hazard named entity recognition task to be solved in this paper.

### Parameter adjustment analysis

The model in this paper is a deep learning method based on a neural network. The neural network randomly provides some hyperparameters to support training, but this will decrease the training efficiency and model performance. We adjust the hyper parameters to obtain the best performance of the model. This paper focuses on the influence of the dropout rate^55^ on the natural hazard named entity recognition model. This paper analyzes the training effects of XLNET-BILSTM-CRF and BILSTM-CRF on NHNC datasets and PFR People's Daily datasets to study the impact of dropout rate on deep learning models. For a fair comparison, all other hyperparameters are left unchanged for the selected best model. Table 6 shows that the F1-score does not increase completely with increasing dropout rate. The F1-score obtained by dropout = 0.75 is lower than that obtained by dropout = 0.5. In both datasets, the performance of both models peaked at dropout = 0.5. It can be seen that the model performs better when dropout is used than when dropout is not used, and the same effect is achieved on different datasets.

**Table 6:** The influence of different dropout on the performance of the model.

Dropout	XLNet-BiLSTM-CRF		BiLSTM-CRF	
	NHNC	PFR	NHNC	PFR
Dropout = 0	90.36	89.87	83.12	83.2
Dropout = 0.25	91.39	90.91	84.82	85.39
Dropout = 0.5	92.27	93.18	86.33	85.84
Dropout = 0.75	92.14	92.29	86.08	85.14

### Text analysis of natural hazard

In text data analysis, previous statistical methods (such as text rank^56^ and TF-IDF^57^) extract only keywords and high-frequency words, not entities. These methods usually need to segment the text data first, then delete the stop words (common words, function words, etc.) to obtain the content words, and finally count the frequency of the content words. However, to better analyze the literature on natural hazards research, we use the method of named entity recognition to extract the knowledge we need, not merely the content words in the natural hazard literature. Through the XLNet-BiLSTM-CRF, we can efficiently extract three types of entities, namely, geographical location, natural hazards and research method, from the research literature related to natural hazards; thus, we can analyze them more intuitively.

In this paper, the research literature related to natural hazards in the last ten years was collected from the Wanfang Database. These data will be recognized by the model proposed in this paper, so as to analyze the research status of natural hazards in recent ten years, and prove that the natural hazard named entity recognition model proposed in this paper has universality. Using the trained XLNet-BILSTM-CRF model to identify the literature, we recognized 1267 geographical locations, 2354 natural hazards and 934 research methods.

**Figure 6:**
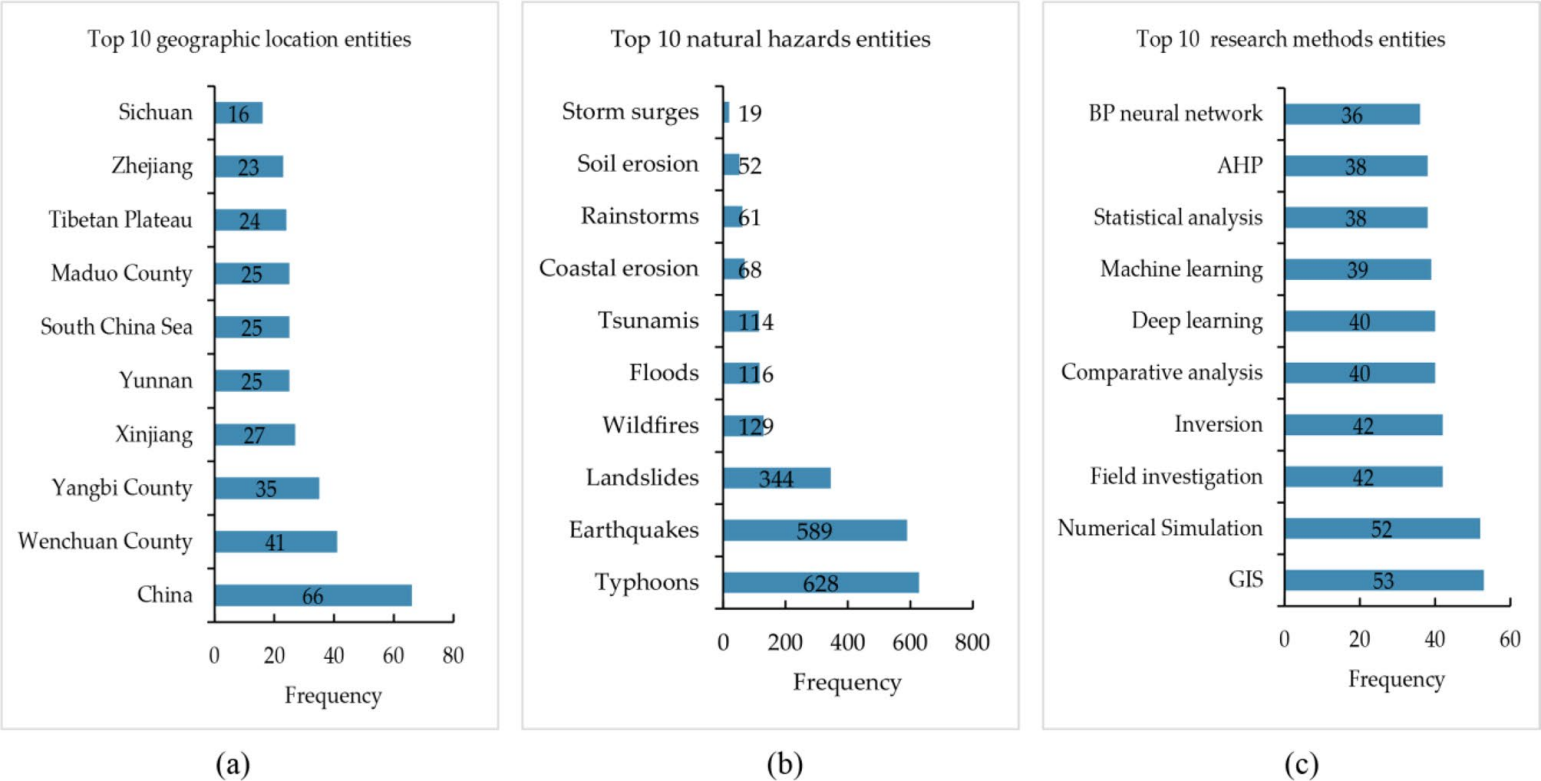
Statistics of natural hazard named entities: (**a**) shows the top 10 geographical location entities among the recognition results, (**b**) shows the top 10 natural hazards entities among the recognition results, (**c**) shows the top 10 research methods entities among the recognition results.

At the same time, according to the recognition results, we counted the most frequent entities with the highest frequency within the geographical location, natural hazards and research method, and the statistical results are shown in Figs. 6. It can be seen that the method proposed in this paper correctly extracts the relevant location and regional descriptions, natural hazards and models and methods used in these natural hazards research papers. This is very helpful for the study, reuse and reference of natural hazards literature.

**Figure 7:**
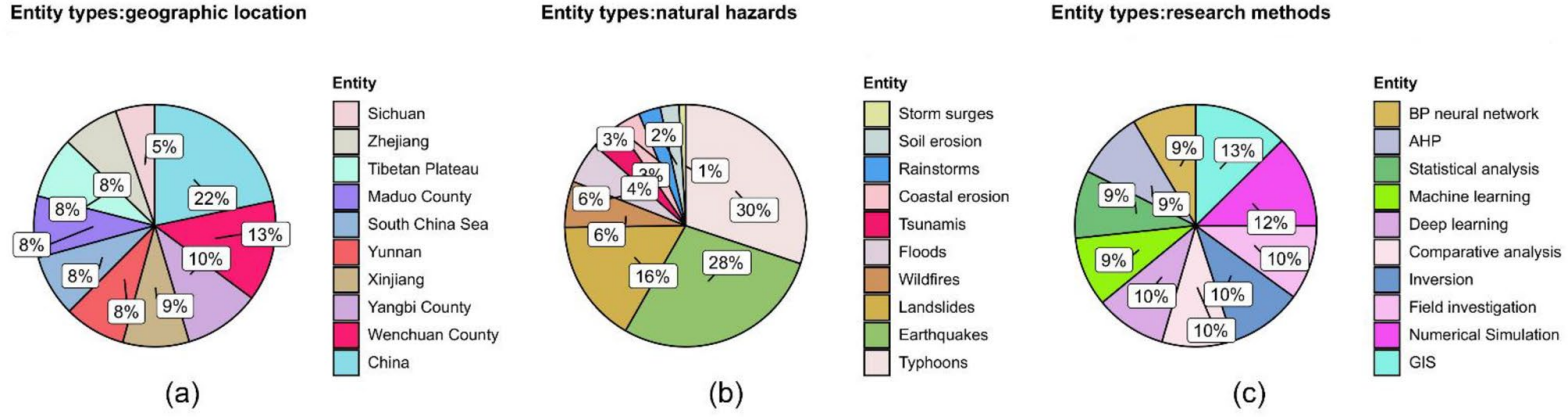
Proportion charts of the three types of entities: (**a**) Proportion of geographical location, (**b**) Proportion of natural hazards, (**c**) Proportion of research methods.

The word frequency of geographical location entities is shown in Fig. 6a. China is the most studied area among geographical location entities, with 66 occurrences, while other regions show a steady downward trend. Figure 7a shows the proportion of geographical entities. The five entities with the highest frequency (China, Wenchuan County, Yangbi County, Xinjiang and Yunnan) account for approximately 62% of the total, and the proportions of other entities are relatively small. Overall, the study of natural hazards covers a wide range of areas, including studies in all parts of China.

The word frequency of natural hazards entities is shown in Fig. 6b. Typhoons, earthquakes and landslides are the main contents for the study of natural hazards, which have quite different frequencies from other natural hazards. The occurrence frequencies are 628, 589 and 344, respectively. Figure 7b shows the proportion of natural hazards, in which three natural hazards entities (typhoons, earthquakes and landslides) account for a total of 74%, and the other seven natural hazard entities account for only 26%.

The word frequency of research methods entities is shown in Fig. 6c. Figure 6c illustrates that among the research methods entities, GIS and numerical simulation are the most widely studied, with frequencies of 53 and 52, respectively, and the gap in the number of research method entities is small. Figure 7c displays the proportions of research method. The entities with high research interest are GIS (13%), numerical simulation (12%), field investigation (10%) and inversion (10%), each of which accounts for more than 10%. The proportion distribution is relatively average.

**Figure 8:**
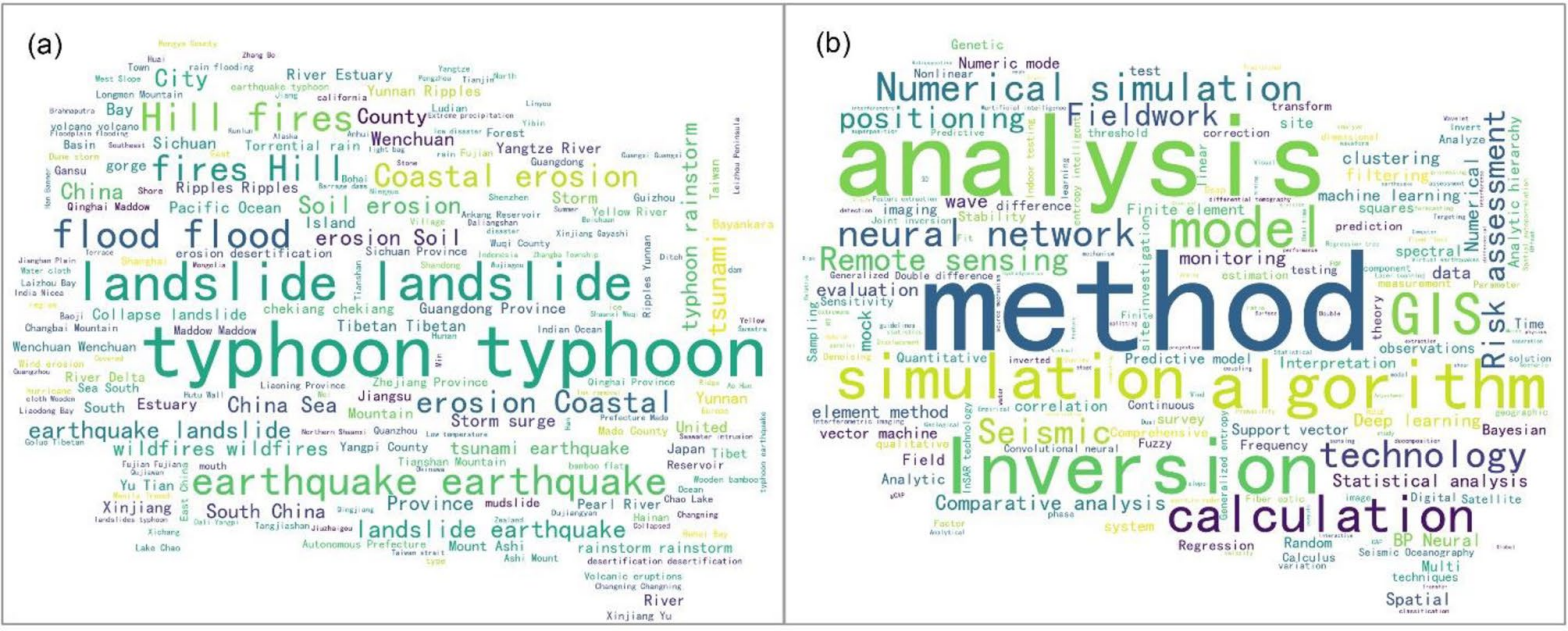
Word cloud of the identification results of the recognition geological structure literature: (**a**) natural hazards and geographical location, (**b**) research method. The larger the font in the word cloud is, the higher the frequency of occurrence. The larger the font in the word cloud is, the higher the frequency of occurrence.

Finally, to visually display the research status of natural hazards by scholars in the past 10 years, we use word clouds to visualize the extracted natural hazards entities. The threshold N for the word cloud is set to 200, meaning that the number of words with high frequency displayed does not exceed 200. The visualization results are shown in Fig. 8.

Figure 8 shows a brief visualization of the results of regional geological structure literature recognition. We can clearly observe the frequent occurrence of natural hazards research in the past decade, such as typhoon, landslide, and earthquake. At the same time, the method proposed in this paper can accurately identify geographical locations in the text. In terms of geographical regions, researchers have covered almost the whole scope of regions in China. Figure 8b shows the complex and diverse research methods, among which the words such as analysis and method have the highest word frequency.

## Conclusions

This paper first classifies natural hazard entities and constructs a natural hazard annotated corpus. In addition, nine named entity recognition methods based on deep learning are used to perform the natural hazard named entity recognition task on the corpus. On the basis of ensuring the practicability of the corpus, the performances of BERT, ALBERT and XLNet pretraining models are compared and analyzed. Then, nine natural hazard named entity recognition methods are combined to analyze and compare their performance from the perspectives of pretraining, feature extraction and decoding. According to the training parameters, the optimal model is then adjusted and trained. Finally, the optimal natural hazard named entity recognition model XLNet-BiLSTM-CRF is selected to recognize the entities in natural hazard papers and identify the research hotspots of natural hazards. This paper draws the following conclusions:

1. XLNet offers the best performance in pretraining, and using BiLSTM as the encoding layer and CRF as the decoding layer can achieve an excellent recognition effect. The precision, recall and F1-score of the XLNET-BILSTM-CRF model were 92.80%, 91.74% and 92.27%, respectively, showing the best performance among the nine models.
2. In the research on natural hazards in the past ten years, northwest China is the main area, and the topics such as "typhoon", "earthquake", "GIS" are the research hotspots.

## Data Availability

The data that support the findings of this study are available from the corresponding author, [H.H.], upon reasonable request.
